# Predictors of relapse after discontinuing systemic treatment in autoimmune chronic uveitis

**DOI:** 10.1186/1546-0096-12-S1-O6

**Published:** 2014-09-17

**Authors:** Gabriele Simonini, Claudia Bracaglia, Marco Cattalini, Andrea Taddio, Alice Brambilla, Cinzia de Libero, Denise Pires Marafon, Roberto Caputo, Rolando Cimaz

**Affiliations:** 1 Rheumatology Unit-Dpt of Padiatrics University of Florence-Anna Meyer Children Hospital, Firenze, Italy; 2 Division of Rheumatology, Department of Paediatric Medicine, IRCCS Ospedale Pediatrico Bambin Gesù, Roma, Italy; 3 Pediatric Clinic, University of Brescia, Brescia, Italy; 4 Department of Sciences of Reproduction and Development, Institute of Child Health, IRCCS Burlo Garofolo, University of Trieste, Trieste, Italy; 5 Ophthalmology Unit, Anna Meyer Children’s Hospital, , Firenze, Italy

## Introduction

Non infectious uveitis in childhood is a relatively uncommon severe disease, with potential significant long-term complications such as cataract, glaucoma and eventually blindness. For these reasons refractory uveitis usually requires early and aggressive immunosuppressive treatment in order to preserve visual acuity and to prevent the significant morbidity of chronic steroid administration. However, the lack of evidence from head-to-head randomized controlled trials (RCT) limits our understanding about the best treatment choices, as well as time of instituting therapy and its duration. Once a child with active uveitis has achieved remission on treatment, there are no evidence-based guidelines with respect to the duration of continued treatment in autoimmune child chronic uveitis. Information regarding the natural clinical history of a child on systemic treatment due to auto-immune chronic uveitis would be helpful in driving therapy: if it would be possible to identify early children at high risk to flare once systemic therapy is stopped, they could have benefits in visual acuity prognosis and quality of life, as well as time free from disease.

## Objectives

Aim of our study was to assess the time on remission after discontinuing systemic therapy in a retrospective, comparative, multi-centre, cohort study of childhood non-infectious chronic uveitis.

## Methods

39 patients (29 F, 10 M; median age: 11.6 years, 31 JIA, 8 Idiopathic Chronic Uveitis [IdCU]) from 4 different paediatric rheumatology centres, with previously refractory, vision threatening, non-infectious *inactive* uveitis, which discontinued all related treatments for at least 3 months were enrolled. 23 children previously received Methotrexate, 16 TNF inhibitors. Primary outcome was to assess, once remission was achieved, the time on remission up to the first relapse after discontinuing treatment. Time to remission once systemic not-steroid treatment was started, time to steroid discontinuation, number of relapses before achieving remission and time on remission on therapy before discontinuing all treatments were also considered.

## Results

Median follow-up time on remission without treatment was 9 months (range 1–59 months). At last available follow-up after 1 year from discontinuation of treatment[49 months, range 15-168], 11/39 (28.2%) children maintained a complete remission over a median period of 18 months. At 49 months of follow-up, 6/8 children with IdCU (75%) compared to 5/31 children with JIA (16.1%) were still on remission without treatment (p<0.003). A higher probability of maintaining uveitis remission after discontinuing treatment was shown in IdCU compared to JIA group (Mantel-Cox c2 7.62, p<0.006). ANA positivity was associated with a higher probability of flare in overall population (Mantel-Cox c2 6.68, p<0.01), in ICU, but not in sub-analysis limited to JIA (Mantel-Cox c2 0.78, p=0.37) and IdCU (Mantel-Cox c2 1.18, p=0.27). None clinical variable, including time on remission on therapy, total length of treatment, and type of treatment, resulted significant predictors of long-lasting remission without therapy.

## Conclusion

Even if limited to a relatively small group in retrospectively study design, our results suggest that type of disease, rather than the type or the length of treatment, can predict different duration of uveitis remission without systemic therapy.

## Disclosure of interest

None declared.

